# Comparison of Glaucoma-Relevant Transcriptomic Datasets Identifies Novel Drug Targets for Retinal Ganglion Cell Neuroprotection

**DOI:** 10.3390/jcm10173938

**Published:** 2021-08-31

**Authors:** Tim J. Enz, James R. Tribble, Pete A. Williams

**Affiliations:** Department of Clinical Neuroscience, Division of Eye and Vision, St. Erik Eye Hospital, Karolinska Institutet, 171 64 Stockholm, Sweden; tim.enz@aol.com (T.J.E.); james.tribble@ki.se (J.R.T.)

**Keywords:** glaucoma, retinal ganglion cells, RNA-sequencing, neuroprotection, drug discovery

## Abstract

Glaucoma is a leading cause of blindness and is characterized by the progressive dysfunction and irreversible death of retinal ganglion cells. We aimed to identify shared differentially expressed genes (DE genes) between different glaucoma relevant models of retinal ganglion cell injury using existing RNA-sequencing data, thereby discovering targets for neuroprotective therapies. A comparison of DE genes from publicly available transcriptomic datasets identified 12 shared DE genes. The Comparative Toxicogenomics Database (CTD) was screened for compounds targeting a significant proportion of the identified DE genes. Forty compounds were identified in the CTD that interact with >50% of these shared DE genes. We next validated this approach by testing select compounds for an effect on retinal ganglion cell survival using a mouse retinal explant model. Folic acid, genistein, SB-431542, valproic acid, and WY-14643 (pirinixic acid) were tested. Folic acid, valproic acid, and WY-14643 demonstrated significant protection against retinal ganglion cell death in this model. The increasing prevalence of open access-omics data presents a resource to discover targets for future therapeutic investigation.

## 1. Introduction

Glaucoma is a common neurodegenerative disease characterized by the progressive dysfunction and loss of retinal ganglion cells (RGCs), the major output neurons of the retina. The disease clinically manifests with deterioration of visual sensitivity and progressive visual field deficits. The major risk factors for glaucoma are age, genetics, and elevated intraocular pressure (IOP). Despite extensive research, IOP management remains the only clinically available therapy and, to date, there are no clinically available neuroprotective strategies for glaucoma. Despite continuing attempts to lower IOP pharmacologically and surgically, a significant percentage of patients ultimately progress to blindness in one or both eyes [1]. Affecting ~80 million patients worldwide, glaucoma is the most common irreversible blinding disease, constituting a substantial health and economic burden [2]. Thus, efficient neuroprotective therapies would be of great value.

The underlying mechanisms of RGC degeneration that lead to glaucomatous optic neuropathy have been studied broadly using a number of animal-based models, of which controlled optic nerve crush [3] or transection [4], bead models of ocular hypertension [5,6], and the DBA/2J mouse model of glaucoma [7] have been commonly used. Although these models vary in their pathology and mechanism of neural insult, some intrinsic cellular degenerative mechanisms and genetic pathways might be inherent to glaucomatous RGC death. Such mechanisms and genetic pathways would be of particular value as targets for therapeutic interventions.

Recently, RNA-sequencing has been utilized to investigate the molecular mechanisms that drive RGC death in these models, allowing for the identification of differentially expressed genes (DE genes) and pathways. Comparing these across models could provide important data for the discovery of common cellular mechanisms and new insights into the pathophysiology of glaucoma. The identified molecules might be targeted by novel or pre-existing pharmaceutical compounds, which would represent a new data-driven approach to discovering potential glaucoma therapeutics, especially when combined with models that allow for rapid testing of drug candidates.

In this study, we identify compounds that could act on altered genes/proteins common to RGC insult using publicly available transcriptomic datasets and validate these using the mouse retinal explant model of RGC degeneration.

## 2. Materials and Methods

### 2.1. RNA-Sequencing Comparison and Identification of Compounds via the Comparative Toxicogenomics Database

Previous results of RNA-sequencing from three different animal models (mouse controlled optic nerve crush [3], DBA/2J mouse model of glaucoma [8], and rat optic nerve transection [4]) were examined. Matrices of DE genes were analyzed at a false discovery rate (FDR, *q*) of 0.05 (0.10 for the mouse controlled optic nerve crush, as reported in the original study). Gene lists were compared in a three-way analysis in R showing a number of shared DE genes between each model. Pathway analysis was performed (Ingenuity pathway analysis, Qiagen, Hilden, Germany) on shared DE genes between comparisons. The shared DE gene list common to all three models was queried in the Comparative Toxicogenomics Database (CTD), which compiles known gene/protein interactions with chemicals based on published data, and compounds targeting a significant proportion of the identified DE genes (>50% occurrence) were identified. Compounds for neuroprotective testing were selected based on previous neuroprotective literature support and novelty to glaucoma.

### 2.2. Animal Strain and Husbandry

All breeding and experimental procedures were performed in accordance with the Association for Research for Vision and Ophthalmology Statement for the Use of Animals in Ophthalmic and Research. Individual study protocols were approved by Stockholm’s Committee for Ethical Animal Research (10389-2018). All mice were housed and fed in a 12 h light/12 h dark cycle with food and drinking water available *ad libitum*. C57BL/6J and B6.Cg-Tg(Thy1-CFP)23Jrs/J (JAX stock number #003710; CFP + RGCs) mouse strains were purchased from The Jackson Laboratory (Bar Harbor, ME, USA) and bred and maintained in-house. All mice were used at 12–20 weeks of age.

### 2.3. Retina Axotomy Explant Model

Compounds were tested using a retinal axotomy model that has been previously described [9]. Mice were euthanized by cervical dislocation, and eyes were enucleated immediately. Subsequently, retinas were dissected free in cold HBSS and either fixed immediately with PFA for 1 h (0 days ex vivo; control) or flat mounted on cell culture inserts (Millicell 0.4 µm pore; Merck, Kenilworth, NJ, USA) and maintained in culture (37 °C, 5% CO_2_) with Neurobasal-A media supplemented by 2 mM L-glutamate (GlutaMAX, Gibco, Thermo Fisher Scientific, Waltham, MA, USA), 1% N-2 supplement, 2% B-27 supplement, and 1% penicillin/streptomycin (all Gibco) in six-well culture plates (3 days ex vivo). For treated retinas, valproic acid (1 mM), folic acid (50 μg/mL), SB-431542 (10 μM), WY-14,463 (100 μM), and genistein (10 μM) were dissolved in the culture media (all drugs from Merck). Half of the media volume was replaced at 48 h. After 72 h (3 days ex vivo), the retinas were removed from culture, fixed in 3.7% PFA for 30 min, and immunolabelled as detailed below.

### 2.4. Immunofluorescent Labelling

Retinas were transferred to slides and isolated using a hydrophobic barrier pen (VWR, Radnor, PA, USA). Subsequently, the tissue was permeabilized with 0.5% Triton X-100 (VWR) in 1 M PBS for 60 min and blocked in 2% bovine serum albumin (Thermo Fisher Scientific) in 1 M PBS for 60 min. Primary antibodies were applied and maintained overnight at 4 °C (detailed in Table 1). Retinas were washed five times for 5 min in 1 M PBS before the secondary antibodies were applied and maintained for 4 h at room temperature. All tissue was washed again five times for 5 min with PBS, and TOPRO-3 nuclear stain (1 µM in 1 M PBS) was applied and maintained at room temperature for 10 min. Tissue was then washed once in PBS and mounted using Fluoromount-G and glass coverslips (Thermo Fisher Scientific). Slides were sealed with nail varnish.

### 2.5. Analysis of Retinal Ganglion Cell Degeneration

RGC loss and shrinkage of nuclei were evaluated by CFP, RBPMS, and TOPRO-3 labelling of flat mounted tissue. All images were acquired on a Leica DMi8 microscope with a CoolLED pE-300white LED-based light source and a Leica DFC7000 T fluorescence color camera (all Leica, Wetzlar, Germany). In each retina, six images (40× magnification, 0.55 NA) were taken at 0, 2, 4, 6, 8, and 10 o′clock equidistantly, about a superior to inferior line through the optic disc (~1000 μm eccentricity). All images were cropped to 0.01 mm^2^. CFP+ cells, RBPMS+ cells, and TO-PRO-3+ nuclei were counted using the cell counter plugin for Fiji [10]. Cell counts and nuclei counts were averaged across the six images in each retina and expressed as a density per 0.01 mm^2^. To assess nuclear shrinkage, the nuclear diameter was measured in >30 nuclei belonging to RBPMS+ cells (per cropped image) using the in-built line tool, giving an average nuclear diameter per image. These values were averaged across the six images of each retina to produce a final average nuclear diameter per retina.

### 2.6. Statistical Analysis

All statistical analysis was performed in R. Data were tested for normality with a *Shapiro Wilk* test. Normally distributed data were analyzed by *Student’s t*-test or *ANOVA* (with *Tukey’s HSD*). Non-normally distributed data were transformed using squared transforms; data that remained non-normally distributed were analyzed by a *Kruskal–Wallis* test followed by *Dunn’s* tests with *Benjamini and Hochberg* correction. Unless otherwise stated, * = *p* < 0.05, ** = *p* < 0.01, *** *p* < 0.001, NS = non-significant (*p* > 0.05). For box plots, the center hinge represents the median, with the upper and lower hinges representing the first and third quartiles; whiskers represent 1.5 times the interquartile range.

## 3. Results

### 3.1. Comparison of Glaucoma-Relevant Transcriptomic Datasets Identifies Common Genes for Therapeutic Targeting

**Figure 1:**
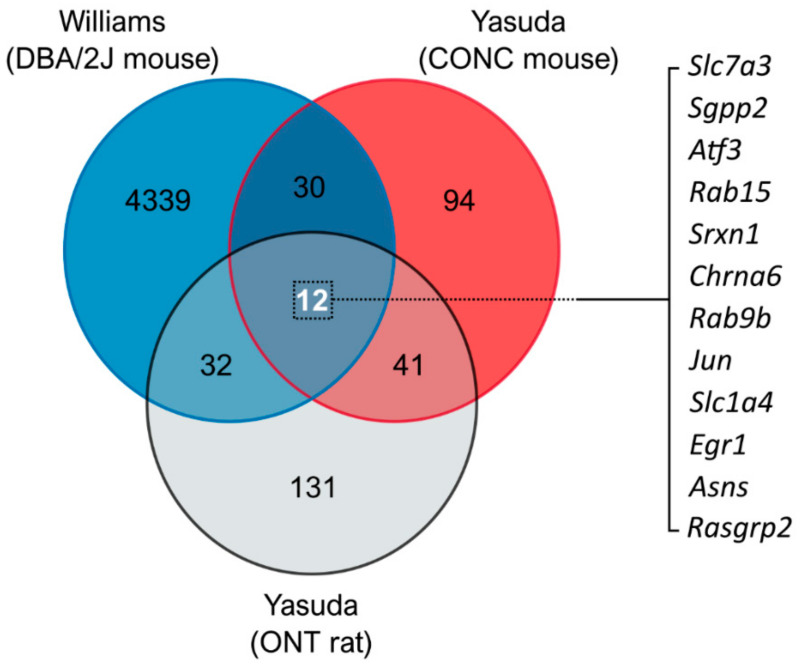
**Comparison of RNA-sequencing data identifies gene changes common to models of retinal ganglion cell injury.** DE gene lists were compiled from publicly available RNA-sequencing datasets from the DBA/2J mouse model of glaucoma (Williams et al., 2017 [8]), mouse controlled optic nerve crush model (CONC; Yasuda et al., 2014 [3]), and rat optic nerve transection model (ONT; Yasuda et al., 2016 [4]). The results are displayed as a Venn diagram showing the total number of DE genes by dataset, and overlap demonstrating shared DE genes. A three-way comparison identified 12 common genes (listed to right) that may represent gene changes conserved to RGC injury, and thus useful therapeutic targets.

Publicly available transcriptomic datasets from three different animal models relevant to glaucoma (mouse controlled optic nerve crush [3], DBA/2J mouse model of glaucoma [8], and rat optic nerve transection [4]) were analyzed to identify common pathways and shared gene expression profiles. Comparison of the DE genes revealed commonality between models with ~30–40 DE genes shared between individual comparisons (comparisons are displayed in Figure 1 and DE gene lists are detailed in Supplementary Table S1). Three-way comparison of differentially expressed genes identified 12 shared DE genes between all three models (Figure 1 and Supplementary Table S1). The identity and role of these genes are detailed in Supplementary Table S2. Pathway analysis revealed a role of these shared DE genes predominantly in the immune system, neuroinflammatory signaling, and amino acid biosynthesis pathways (Table 2). However, the majority of pathways had a low number of gene hits (pathway hit %), demonstrating that these identified DE genes do not collectively belong to a single or conserved pathway of RGC degeneration.

We then used the CTD to screen for compounds that interact with these shared DE genes in order to identify potential novel therapeutics. Screening revealed 40 compounds that interact with >50% of these shared genes (Supplementary Table S3). A number of these are chemical by-products or inorganic compounds tested in toxicity assays (e.g., for carcinogenic effects) and as such are not suitable therapeutics. Other compounds had known neurodegenerative or anti-neuroprotective properties/responses (e.g., LPS and genistein). We identified eight compounds that may be suitable therapeutics based on a literature search (Supplementary Table S3), as they are either hormonal compounds, simple dietary compounds, or compounds that have already been tested in neurodegenerative contexts. These eight compounds were valproic acid, SB-431542 (an inhibitor of TGF-Beta Type I Receptor/ALK5, ALK4 & ALK7), progesterone, estradiol, choline, folic acid, WY-14643 (pirinixic acid, a peroxisome proliferator-activated receptor alpha (PPARα) agonist), and rosiglitazone.

### 3.2. Retinal Explant Model Provides a Platform to Rapidly Test Candidate Neuroprotective Therapeutics

**Figure 2:**
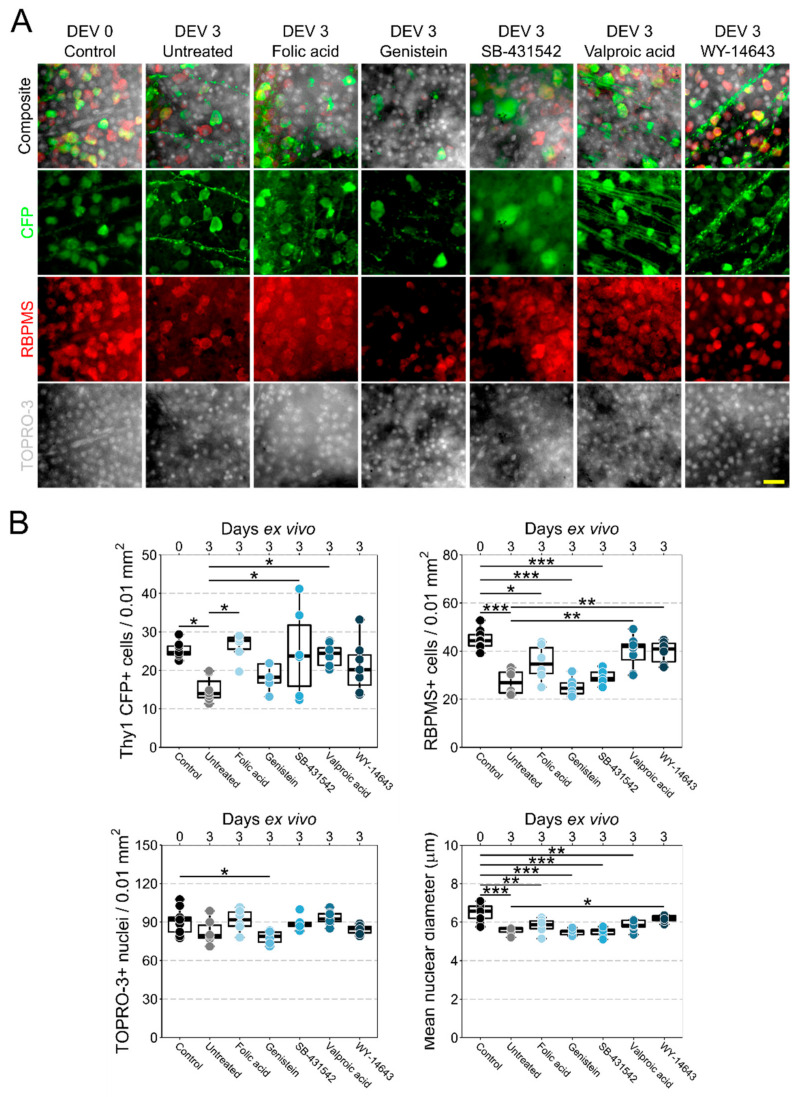
**Sequencing data and drug database screening successfully identifies therapeutic candidates that provide retinal ganglion cell neuroprotection.** Retina from B6.Cg-Tg(Thy1-CFP)23Jrs/J mice were explanted into tissue culture and maintained for 3 days ex vivo (DEV) with candidate drugs supplemented in the media (in addition to controls: 0 DEV and 3 DEV untreated). (**A**) Retinas were labelled for CFP (anti-GFP), RBPMS, and TOPRO-3 before imaging. (**B**) CFP+ cell density was significantly reduced at 3 DEV in untreated retinas and this was significantly improved in folic acid, SB-431542, and valproic acid treated retinas. RBPMS+ RGC density was significantly reduced at 3 DEV in untreated retinas and this was significantly improved in valproic acid and WY-14643 treated retinas, with moderate protection from folic acid. TOPRO-3+ round nuclei were not significantly altered with the exception of genistein, supporting its lack of protection and possible neurotoxic effects. Mean nuclear diameter was significantly smaller at 3 DEV, and this was significantly improved by WY-14643. Overall, these data support valproic acid, folic acid, and WY-14643 as neuroprotective against acute RGC injury. This validates the approach of identifying potential neuroprotective therapeutics from existing -omics data. Scale bar in A = 20 µm. * *p* < 0.05, ** *p* < 0.01, *** *p* < 0.001.

Axotomy of the RGC axon results in RGC degeneration. In the retina explant model [9,11], severing of the optic nerve leads to RGC axotomy, and results in 30–50% RGC loss over 3–5 days in the mouse. We utilized this model to identify compounds for an RGC neuroprotective effect (Figure 2). Valproic acid, SB-431542, folic acid, and WY-14643 were selected for testing because an established literature already exists for progesterone, choline, and estradiol (see discussion). Following axotomy and maintenance in culture ex vivo for 3 days, retinas exhibited a marked loss of RGCs as identified by significant reduction in the number of CFP+ cells (40% loss, *p* < 0.05) and RBPMS+ cells (39% loss, *p* < 0.001). Nuclear density was variable and was not significantly reduced at this time point, likely reflecting a combination of neuronal loss and glial proliferation. Surviving RGCs had significantly reduced nuclear diameter (14% smaller, *p* < 0.001), indicating significant cellular stress.

Three of the tested compounds conferred significant neuroprotection when dissolved in the culture media. Survival of CFP+ RGCs was best promoted by folic acid (1.75-fold survival from untreated, *p* < 0.05), followed by valproic acid (1.59-fold survival from untreated, *p* < 0.05), and was significant, but highly variable with SB-341542 (1.65-fold survival from untreated, *p* < 0.05). RBPMS+ cell counts were highest in retinas treated with valproic acid (1.47-fold survival from untreated, *p* < 0.01; 11% loss compared with control, *p* > 0.05), followed by WY-14643 (1.4-fold survival from untreated, *p* < 0.01; 11% loss compared with control, *p* > 0.05). RGC loss assessed by RBPMS+ counts was reduced for folic acid treated retinas relative to control (21% loss compared with control, *p* > 0.05), but was not significantly different from untreated retinas, demonstrating variable protection (1.3 fold survival from untreated, *p* > 0.05). Only WY-14643 demonstrated significant protection against nuclear shrinkage (1.11-fold larger diameter compared with untreated, *p* < 0.05; 4.5% loss compared with control, *p* < 0.05). Folic acid and valproic acid demonstrated a less severe nuclear shrinkage relative to control (10% loss, *p* < 0.01; and 9.7% loss, *p* < 0.01, respectively). SB-341542, despite showing some protection to CFP+ RGCs, did not demonstrate significant neuroprotection to RBPMS+ RGCs (1.08-fold survival from untreated, *p* > 0.05; 35% loss compared with control, *p* > 0.001), or against nuclear shrinkage (0.98-fold survival from untreated, *p* > 0.05; 15% loss compared with control, *p* > 0.001), suggestive of a possible RGC subtype bias or preferential protection of healthier cells (i.e., those able to maintain CFP expression) [12].

As further validation of this approach, we selected an identified compound that should not enhance RGC survival (as the CTD returns interactions based only on literature link, irrespective of context). We tested the effects of supplementing the culture media with genistein as it has been demonstrated to influence a number of neuroprotective effects. Genistein had no effect on RGC survival compared with untreated retinas, as assessed by CFP+ counts (*p* > 0.05), RBPMS+ counts (*p* > 0.05), TOPRO-3+ counts (*p* > 0.05), or nuclear diameter (*p* > 0.05). Density of TOPRO-3+ nuclei was actually significantly reduced from control in only genistein-treated retinas (15% loss from control, *p* < 0.05).

## 4. Discussion

The key defining characteristic of glaucomatous optic neuropathy is the progressive loss and dysfunction of RGCs and is one of the only shared features across the pathophysiological spectrum in human glaucomatous disease and animal models of glaucoma. The pathophysiology of RGC degeneration in different animal models is likely to vary. There may be shared intrinsic RGC degenerative mechanisms across all glaucoma models as well as human glaucoma, which can be identified and explored. The purpose of this study was to identify commonalities between RGC injury models (rather than to individually analyze distinct models of glaucoma) with which to identify neuroprotective treatments that may be applicable across the heterogeneity of glaucoma (in animal models and human glaucoma patients).

In this study, we used three publicly available RNA-sequencing datasets of animal models of glaucoma. The DBA/2J mouse is one of the most frequently used glaucoma models in research. In DBA/2J mice, mutations in two genes (*Gpnmb^R150X^* and *Tyrp1^b^*) drive an iris disease with features of human iris atrophy and pigment dispersion. Pigment disperses from the iris and induces damage in the drainage structures of the eye. This inhibits aqueous humor outflow and leads to an increase in IOP. By 9 months of age, IOP is high in the majority of eyes and transcriptomic and metabolic datasets demonstrate mitochondrial and metabolic dysfunction in RGCs [12]. In the mouse controlled optic nerve crush model, axonal injury is induced mechanically by a temporary compression of the distal optic nerve, resulting in RGC death [3]. Similarly, in the optic nerve transection model, axonal injury triggers axon degeneration, leading to a rapid RGC degeneration [4].

With the increased prevalence of -omics technologies, there is a wealth of open data within the field of glaucoma for researchers to explore and utilize. The aim of the present study was to unbiasedly test a conserved gene set between three publicly available RNA-sequencing datasets. Typically, pathway analysis and ranking of changed genes/proteins/metabolites reveal multiple potential mechanistic avenues for exploration, but practical (funding and publication limits) and narrative/hypothesis limitations leave many of these unexplored. Comparison of datasets to identify common changes can be a powerful method to identify conserved pathological processes in optic nerve injury, as has been demonstrated by previous comprehensive comparisons including the DBA/2J mouse model, optic nerve crush/axotomy models across multiple species, and other CNS neurodegenerations [13,14]. These studies identified commonality and enrichment predominantly within neuroinflammatory and innate immune responses. With the growth of this approach, the field can form data-driven hypotheses to identify and test new mechanisms of neurodegeneration/neuroprotection.

In this study, we compared common gene changes in transcriptomic datasets from RGC injury models and screened these against the Comparative Toxicogenomics Database to identify novel therapeutics. Using a retinal explant model to rapidly test a number of these compounds, we demonstrate the therapeutic potential of drugs identified in this way. Multiple drugs identified had established literature support for RGC and neuronal protection, further supporting this method of identifying potential therapeutics. The ex vivo retinal explant model is ideal as a first-pass model for assessing neurodegenerative events and testing neurodegenerative or neuroprotective drug candidates. In this model, RGCs are axotomized, leading to RGC degeneration [9,11]. As 100% of RGCs are axotomized, the insult is controlled, and maintenance in tissue culture removes the influence of systemic events (e.g., infiltration of myeloid-derived cells) and tightly controls tissue conditions. Only small amounts of a drug is required for testing as it can be directly dissolved in the media. This overcomes any need to test or assess systemic metabolism and bioavailability in the first round of studies, thus keeping the cost of drug and animals low. Further to this, in many research-centric countries (e.g., EU, USA, Australia), additional animal ethical permits are not required to perform this model, increasing its availability to labs that might not have significant animal housing and testing resources.

Screening the shared DE genes between the three glaucoma models against the Comparative Toxicogenomics Database identified valproic acid as the drug that interacted with the most gene products (11/12; the exception was *Chrna6*), followed by SB-431542, progesterone, estradiol, choline, folic acid, WY-14643, and rosiglitazone (see Supplementary Table S3). Valproic acid is an FDA-approved anti-epileptic and migraine drug. Valproic acid’s mechanism of action is proposed to be through modulating histone deacetylases activity, which has a well-established literature of limiting RGC degeneration in models of normal-tension and ocular-hypertensive glaucoma [15,16].

Progesterone and estradiol are both endogenous steroids and sex hormones. An extensive literature exists on their possible potential as neuroprotectors and multiple different mechanisms of action are proposed (e.g., regulation of mitochondrial calcium and *Bcl-2* expression, affecting the phosphatidylinositol 3-kinase/Akt signal pathway or preventing caspase-3 activation). A number of studies have been performed to explore the potential of progesterone and estradiol as neuroprotectors in animal models of photoreceptor and retinal ganglion cell loss [17,18,19,20,21,22,23,24,25]. Given the evidence that RGCs express estrogen receptors, lower estradiol levels are linked with primary open-angle glaucoma (POAG) [26], and that β-estradiol can protect from RGC death [23,27,28], an estrogen analogue-based therapy may be of benefit in many glaucoma patients.

Choline is a quaternary amine, the precursor of many cell components and signaling molecules (e.g., acetylcholine), and an essential nutrient for humans. It is further processed to citicholine and phosphatidylcholine in vivo. Both choline and citicoline are thought to have a neuroprotective effect, possibly by preserving sphingomyelin and cardiolipin and by promoting glutathione synthesis. The literature contains a number of studies suggesting a favorable effect on retinal cell survival, as assessed in various neurodegenerative animal models [15,16,17,18,19].

Folic acid is an essential nutrient and B vitamin which plays a crucial role in the biosynthesis of DNA and RNA. The vitamin is also indispensable for erythrocyte maturation. Clinically, folic acid is mainly used to prevent neural tube defects in the developing fetus. Yet, it has been shown to be protective against certain dysplasia and to reduce gingival inflammation [29]. A number of studies examined the effect of folic acid on microglia and astrocytes in animal models of cellular stress responses, and reported positive results [30,31]. However, to the best of our knowledge, a possible neuroprotective effect in glaucoma has not been previously investigated.

As an established antidiabetic drug of the thiazolidinedione class, Rosiglitazone functions as an insulin sensitizer. The drug binds to the peroxisome proliferator-activated receptors in adipocytes and renders the cells more responsive to insulin. The neuroprotective effect of Rosiglitazone and other thiazolidinediones has been studied extensively and a beneficial effect has been suggested through inhibition of inflammatory responses, pro-apoptotic cascades, and mitochondrial metamorphosis [32,33,34].

The TGF-β type 1 receptor inhibitor SB-431542 is a drug candidate proposed for the treatment of osteosarcomas in humans. The agent acts through binding the activin receptor-like kinase (ALK) receptors ALK5, ALK4, and ALK7. It has also been shown to promote the transformation of astrocytes into neurons [35]. Two studies found evidence for a possible protective effect from NMDA-induced retinal degeneration in rats and from N-methyl-N-nitrosourea (MNU)-induced rod photoreceptor degeneration in zebrafish [36,37].

Pirinixic acid, also known as WY-14643, is a synthetic drug candidate for prevention of severe cardiac dysfunction, cardiomyopathy, and heart failure as a result of lipid accumulation within cardiac myocytes. Its mechanism of action is through binding the peroxisome proliferator-activated receptor alpha (PPARα), thereby affecting cell proliferation and differentiation, lipid metabolism, and inflammatory signaling cascades. WY-14463 has the potential to protect neurons by modulating mitochondrial fusion and fission [38]. However, the drug′s potential neuroprotective effect has not previously been explored in glaucoma models. In summary, our screen identified a number of neuroprotective compounds novel to RGC survival. Valproic acid, folic acid, and WY-14643 performed best in terms of neuroprotection, while treatment with SB-431542 led to variable results.

As a further test of this drug discovery method, we sought to also test identified drugs that, based on literature, would not produce a neuroprotective effect. Genistein is a naturally occurring phytoestrogen and isoflavone. The compound is found in soybeans, flava beans, coffee beans, and others. Genistein is known to inhibit protein-tyrosine kinase and topoisomerase-II, affecting the process of cell differentiation and proliferation, and promoting DNA fragmentation and apoptosis [39,40]. It has been hypothesized that genistein could be used to treat different types of cancer through its antioxidant and antiangiogenetic effects. However, in various animal neurodegenerative models, genistein has been shown to block the neuroprotective effect of agents such as carbamylcholine, forskolin, and veratridine [41,42,43]. In the explant model, genistein had no effect on RGC survival, thus supporting the applicability of this method for identifying disease modifying compounds.

## 5. Conclusions

Glaucoma is a complex neurodegenerative disease in which the only shared and clinically defined feature is the progressive dysfunction and degeneration of RGCs (although age, genetic risk, and high IOP are all common risk factors, glaucoma can still occur in the absence of one or more of these risk factors). Many models of glaucoma-related stress have been developed in a wide array of animal species, which recapitulate some of the features of human glaucoma. Comparison of these models to identify common changes can be a powerful method to identify conserved pathological processes in RGC injury. Publicly available -omics datasets such as those from RNA-sequencing represent a data-rich resource for identifying these potential critical pathogenic changes. We identified gene changes common to three modes of RGC injury and screened these against the Comparative Toxicogenomics Database to identify novel therapeutic agents for testing. We demonstrated the validity of this approach by testing the identified compounds using another independent model of RGC injury.

Our drug discovery and analysis platform used only publicly available tools and datasets and an ex vivo model widely amenable to the majority of research-intensive countries that does not require additional animal ethical permits, while keeping drug treatment costs low. This platform is a practical means to utilize the increasing wealth of open access -omics data generated by the glaucoma field in order to move forward towards identifying new therapeutics.

## Figures and Tables

**Table 1:** Antibodies used.

Antibody	Target	Host	Stock Conc.	Working Conc.	Dilution	Details
anti-RBPMS	RNA-binding protein, RGC specific in the retina	Rabbit	660 µg/mL	1.32 µg/mL	1:500	Novusbio NBP2-20112
anti-GFP	XFPs (e.g., CFP)	Chicken	10 mg/mL	20 µg/mL	1:500	Abcam ab13970
Goat-anti Rabbit Alexa Fluor 568	Rabbit primary antibody	Goat	2 mg/mL	4 µg/mL	1:500	Invitrogen A11011
Goat-anti Chick Alexa Fluor 488	Chick primary antibody	Goat	2 mg/mL	4 µg/mL	1:500	Invitrogen A11039

**Table 2:** Pathway analysis of shared differentially expressed genes between RNA-sequencing experiments.

	Ingenuity Canonical Pathways	−log(*p*) *	Molecules	Pathway Hits (%)
**D2 ^†^ vs. Mouse CONC**	Complement System	6.22	ITGAM,C1QA,C1QC,C1QB	10.8
	Neuroinflammation Signaling Pathway	5.87	JUN,AGER,TYROBP,HLA-A,KCNJ5,ATF4,CX3CR1	2.25
	Dendritic Cell Maturation	4.59	TYROBP,HLA-A,FCER1G,ATF4,PLCB1	2.59
	PI3K Signaling in B Lymphocytes	4.04	ATF3,JUN,ATF4,PLCB1	3.08
	GNRH Signaling	3.87	JUN,EGR1,ATF4,PLCB1	2.78
	Role of NFAT in Regulation of the Immune Response	3.44	JUN,HLA-A,FCER1G,PLCB1	2.15
	OX40 Signaling Pathway	3.21	JUN,HLA-A,FCER1G	3.3
	Type I Diabetes Mellitus Signaling	2.96	HLA-A,FCER1G,PTPRN	2.7
	Cytotoxic T Lymphocyte-Mediated Apoptosis of Target Cells	2.81	HLA-A,FCER1G	6.25
	Phagosome Formation	2.77	ITGAM,FCER1G,PLCB1	2.31
**D2 ^†^ vs. Rat ONT**	Glutamate Receptor Signaling	3.72	GRIN2A,SLC1A4,GRIK3	5.26
	Amyotrophic Lateral Sclerosis Signaling	2.87	PRPH,GRIN2A,GRIK3	2.7
	Asparagine Biosynthesis I	2.71	ASNS	100
	MIF Regulation of Innate Immunity	2.49	JUN,PLA2G5	4.65
	Serotonin Receptor Signaling	2.49	HTR5A,HTR1D	4.65
	ATM Signaling	1.8	JUN,GADD45G	2.04
	Sphingosine and Sphingosine-1-Phosphate Metabolism	1.76	SGPP2	11.1
	IGF-1 Signaling	1.73	JUN,YWHAH	1.89
	p53 Signaling	1.7	JUN,GADD45G	1.8
	Neuroinflammation Signaling Pathway	1.64	GRIN2A,JUN,PLA2G5	0.965
**Mouse CONC vs. Rat ONT**	AMPK Signaling	2.76	RAB9B,CHRNA6,CDKN1A,CHRNB3	1.85
	Asparagine Biosynthesis I	2.62	ASNS	100
	PI3K Signaling in B Lymphocytes	2.43	ATF3,JUN,ATF5	2.31
	CXCR4 Signaling	2.14	RHOQ,JUN,EGR1	1.82
	eNOS Signaling	2.09	CHRNA6,SLC7A1,CHRNB3	1.74
	Heme Degradation	2.02	HMOX1	25
	IL-8 Signaling	1.93	HMOX1,RHOQ,JUN	1.52
	IL-10 Signaling	1.93	HMOX1,JUN	2.9
	Tetrahydrofolate Salvage from 5,10-Methenyltetrahydrofolate	1.93	MTHFD2	20
	Serine Biosynthesis	1.93	PHGDH	1.85
**Three-way comparison**	Asparagine Biosynthesis I	3.25	ASNS	100
	PI3K Signaling in B Lymphocytes	2.63	ATF3,JUN	1.54
	GNRH Signaling	2.55	JUN,EGR1	1.39
	CXCR4 Signaling	2.43	JUN,EGR1	1.21
	B Cell Receptor Signaling	2.31	JUN,EGR1	1.05
	Sphingosine and Sphingosine-1-Phosphate Metabolism	2.3	SGPP2	11.1
	AMPK Signaling	2.2	RAB9B,CHRNA6	0.926
	IL-17A Signaling in Gastric Cells	1.86	JUN	4
	TNFR2 Signaling	1.78	JUN	3.33
	4-1BB Signaling in T Lymphocytes	1.75	JUN	3.12

* −log(*p*) > 1.301029996 = *p* < 0.05; ^†^ D2 = DBA/2J.

## Data Availability

All data generated or analyzed during this study are included in this published article.

## Supplementary Materials

The following are available online at https://www.mdpi.com/article/10.3390/jcm10173938/s1, Table S1: Shared differentially expressed genes between glaucoma RNA-sequencing datasets, Table S2: Roles of shared differentially expressed genes, Table S3: Compounds with known interactions with differentially expressed genes common to all three RNA-sequencing datasets.
